# Integrating intercultural communicative competence into an online EFL classroom: an empirical study of a secondary school in Thailand

**DOI:** 10.1186/s40862-022-00174-1

**Published:** 2023-02-01

**Authors:** Tzu-Yin Lee, Yun-Chi Ho, Che-Han Chen

**Affiliations:** 1 Department of English Language and Literature, Tzu Chi University, Hualien, Taiwan; 2 Institute of Education, Tzu Chi University, Hualien, Taiwan; 3 Department of Applied Linguistics and ESL, Georgia State University, Atlanta, GA USA

**Keywords:** Intercultural communicative competence, L2 motivation, English language learning, Quasi-experimental design, Teenage EFL learners

## Abstract

Intercultural communicative competence (ICC) has been widely acknowledged as a core element of today’s foreign language education. However, even though the importance of intercultural language teaching is commonly recognized among adult learners and at the post-secondary level, teachers of adolescent English learners often find it hard to effectively incorporate culture into English learning because of the lack of an instructional model facilitating their students’ intercultural development and English learning experiences. Therefore, this study aims to investigate whether integrating intercultural learning into an online EFL curriculum can elevate teenage EFL students’ L2 motivation, intercultural communicative competence, and English proficiency. The researchers used a quasi-experimental design by randomly selecting two eighth-grade classes in a secondary school in northern Thailand, with one class designated as the experimental group (N = 31) and the other as the control group (N = 28). The effects of this teaching experiment were then examined using both quantitative and qualitative data. The findings demonstrated that the students in the experimental group showed a greater improvement compared with those of the students in the control group after an 8-week, interculturally embedded English curriculum. The results suggested that ICC is conducive to adolescent EFL students’ intercultural development as well as their English learning motivation and outcome. The applications of ICC-based EFL instruction in similar contexts are discussed.

## Introduction

A recent report entitled “Language Policy and Education in Southeast Asia” highlights the growing influence of Southeast Asian countries on today’s globalized economy and interconnected world (Kosonen, 2017). This phenomenon necessitates a more rigorous English language education among Southeast Asian countries’ educational systems to equip more English as a foreign language (EFL) learners with the kind of English ability to effectively and appropriately interact and communicate with people from diverse cultural and linguistic backgrounds (Kirkpatrick, 2012). Under such learning needs and social expectations, many countries in the region have been aspiring to introduce the kind of English teaching methodologies that enable learners to follow native speakers’ linguistic norms and uses to develop near-native English proficiency (Teng & Sinwongsuwat, 2014; van Goidtsnoven, 2019). Communicative language teaching (CLT) has been used by many Southeast Asian countries to achieve this goal, and native-like language competence is a crucial element in the success of this kind of instruction (Butler, 2017; Deerajviset, 2014; Kustati, 2013).

However, the objective of achieving native-like proficiency as the only goal for second language learning is problematic; this would not only prevent students from developing competent language ability, but also acquiring adequate intercultural sensitivity that would enable them to communicate with people speaking different varieties of English and with diverse cultural backgrounds (Weber & Horner, 2012; Wright, 2010). In addition, the context of globalization has necessitated more emphasis on including intercultural communicative competence (ICC) in English language education to “equip learners with the knowledge of intercultural communication and the ability to use it effectively to bridge cultural differences and achieve more harmonious, productive relations” (Tran & Duong, 2018, p. 1). In other words, simply endowing students with native-like linguistic proficiency is unrealistic because the notion of English as an international language denotes the need for connecting English language learning with a variety of cultural input that characterize today’s communicative and multicultural situations (Clark, 2013). Given that ICC stands as the key competence in contemporary foreign language education, it is widely agreed that the ultimate goal of foreign language curricula is to equip students with the ability to function as linguistically and interculturally competent English speakers who can take part in complex and multicultural settings (Chao, 2014; Liu, 2017).

Nevertheless, although the integration of culture into English language learning is well-acknowledged among adult learners and at the post-secondary level (e.g., Chao, 2014; Liu, 2017; Tran & Duong, 2018), scant prior research has investigated how teachers can help teenage EFL students’ elevate their awareness and motivation to connect cultural knowledge with English learning arising from the lack of instructional models to facilitate their intercultural development and English learning experiences. Sompakdee et al. (2021) suggest that the disruption caused by the outbreak of the COVID-19 pandemic seriously limits EFL students’ access to quality English language and intercultural learning because of the unfamiliarity with employing technology or digitally mediated educational tools, which further exacerbates students’ overall motivation in second language learning and achievement. To address this issue, we created an online intercultural experiential English curriculum to engage teenage EFL students. This paper aims to investigate whether utilizing an ICC-embedded English curriculum would stimulate EFL secondary school students’ L2 motivations for and attitudes toward learning about foreign cultures. To evaluate the effectiveness of this curriculum design, three research questions were investigated:

1. Does the ICC-based English curriculum affect adolescent EFL learners’ L2 motivation?
2. Does the ICC-based English curriculum help adolescent EFL learners develop intercultural competence?
3. Does the ICC-based English curriculum increase adolescent EFL learners’ language competence?

## Literature review

### ICC in foreign language education

Given the complex nature of what it means to be an intercultural speaker, applied linguists hold divergent views about its definition, which has often led to confusion and contradictory conceptualizations in assessing them in educational settings (Sincicrope et al., 2007; Tran & Duong, 2018). Byram (1997) asserts that ICC refers to the ability to use a second language (L2) to interact successfully with someone from different cultural and linguistic backgrounds. ICC can also be broadly characterized as having the capability to use effective and appropriate linguistic and paralinguistic strategies to achieve the goal of communication between speakers of different languages. Since the successful use of a second language for communication usually occur between speakers with different cultural backgrounds, Wilson (1986) proposes the concept of “cross-cultural experiential learning,” which means that language learners should put themselves in an environment where they are exposed to diverse intercultural inputs. Such environment would help students become interculturally aware of their own culture and the presence of otherness as well as to respect the differences among them. As a result, both “ICC” and “cross-cultural communication” will be used interchangeably in the following discussions of this study.

With regard to the role of ICC in language education, Byram et al. (2002) contend that the main aim of language teaching should focus on helping learners to act as intercultural speakers or mediators that possess the following two competencies: linguistic competence that are needed to formulate what they want to express in correct and appropriate ways; intercultural competence that prepare learners “to understand and accept people from other cultures as individuals with distinctive perspectives, values, and behaviors, and to help them see such interaction is an enriching experiences.” (Byram et al., 2002, p. 10). In line with this, Fantini (2000, 2001) devises a model of ICC that includes the constructs of awareness, attitudes, skills, and knowledge that are essential to make learners become able to understand and empathize with others on a local and global level. Although Fantini did not explicitly specify linguistic competence as one of the constructs in the ICC model, he stressed that proficiency in both first language (L1) and L2 is a crucial element in the development and acquisition of ICC.

Both Fantini and Byram’s conceptual frameworks draw attention to the need to integrate culture into second language teaching, shedding light on how teachers should define and assess the constructs of intercultural competence. Specifically, drawing on Fantini and Byram’s definition of ICC, Peng et al. (2009) investigated Taiwanese high school students’ intercultural learning and adapted these constructs into a rating scale that can be used to measure adolescent EFL student’s intercultural competence as follows:

1. Awareness: Ability to notice and evaluate the difference between one’s own culture and other cultures.
2. Attitude: Curiosity and degree of openness toward other cultures and beliefs about one’s own.
3. Skills: Ability to interpret and acquire new knowledge of a culture using a second language in real-world settings.
4. Knowledge: The degree of understanding of the similarities and differences between cultures.

With regard to how ICC can be developed in the educational settings, researchers conceptualize cross-cultural experiential learning as a truly meaningful way to acquire intercultural competence because it requires learners to use the target language as a communication tool to critically contemplate their own culture and the taken-for-granted value systems through which they make judgments about the things that surround them (Chao, 2014; Wilson, 1986; Yang, 2017). Byram et al. (2002) suggest two ways for language teachers to help students develop intercultural competence. Classroom-based instruction can be used to train learners to acquire intercultural awareness and knowledge concerning other cultures and different forms of communication, reflect on their own communication skills, and adjust intercultural attitudes by exposing them to authentic tasks or activities such as simulations or role-play under the teacher’s guidance. The other way to develop ICC is through participation in intercultural exchanges or study abroad programs, in which learners can not only activate their schemata about different countries or cultures through in-person contact with the cultural materials (awareness and attitude), but also increase their self-efficacy and confidence regarding their actual implementation of communication skills with the target language in real-life intercultural settings (skills and knowledge). While some studies have shown that the latter method is more capable of helping students increase knowledge, improve language proficiency, and develop cultural sensitivity (Baker, 2011; House, 2012; Young & Sachdev, 2011), other research has illustrated that the former classroom-based pedagogies are more feasible in the majority of EFL settings accommodating the kind classroom cultures (Hu, 2010).

As for the research that have empirically examined the effectiveness of incorporating ICC in the EFL classroom, Liu (2017) integrates experiential learning with intercultural language teaching in an EFL class consisting of 33 non-English major at a Taiwanese university. In this one-semester experiential intercultural English curriculum, the researcher invited multiple international students as the guest speakers to promote the learning of culture and English through real interactions between the guest speakers with EFL students. Based on the course evaluation survey and students’ written products and in-class presentations, students’ intercultural competence is improved in terms of more reflective awareness of how to use English language in intercultural communicative settings. This finding is consistent with Tran and Duong’s (2018) study, which investigates the effectiveness of an intercultural communicative language teaching (ICLT) model in an EFL context. The participants were forty-seven adult Vietnamese EFL learners. After this 13-week curriculum where students deeply explore intercultural materials through trying out different forms of English language and communication strategies, the results show that students show a similar degree of improvement in their intercultural as well as English competence. In sum, these studies have empirically shown that based on in-depth observation and critical reflection through experiential learning, EFL learners not only have greater chances of acquiring intercultural competence in the form of awareness and empowerment of attitudes, knowledge, skills, but also language competence when interacting with people of other languages and cultures through English as an international language (Fantini, 2000).

### Issues in incorporating intercultural language teaching in EFL classrooms

In most EFL contexts, foreign language classes are the most important venues for learners to access opportunities to develop their intercultural learning experiences and competence. However, research has demonstrated that this can be problematic for four main factors. First, Chlopek (2008) and Yeh (2009) note that due to the lack of experiential learning and target language community in a culturally homogeneous classroom, it can be difficult for learners to acquire ICC, particularly in EFL contexts. Furthermore, ineffective pedagogical approaches, lack of appropriate materials, and shortage of competent teachers are other issues hindering the development of ICC among EFL students (Cheng, 2012). In most EFL settings, although most English teachers are increasingly become aware of integrating cultural knowledge into their syllabi, ineffective pedagogies such as linguistics- and accuracy-based teaching still dominate language classrooms as teachers lack instructional models to implement cultural activities with the students (Liu, 2017; Maliwat, 2021; Tran & Duong, 2018). The lack of diverse cultural materials in textbooks and experiential learning may also lead EFL learners to become uninterested in learning and incapable of using English effectively when communicating with others from different linguistic and cultural backgrounds, thereby decreasing their ICC development (Chao, 2011). These observations are consistent with Cheng’s (2012) study, which investigates the influences of university EFL instructors’ understanding of intercultural instruction on their teaching practices in Taiwan. Cheng’s close examinations of the textbooks used in this classroom environment present four major concerns and inadequacy of cultural learning in English classrooms: (1) Western cultural norms, (2) dominance of North American cultures, (3) emphasis on language skills, and (4) lack of cross-cultural awareness activities. This research demonstrates that more focus should be placed on including comparisons and contrasts between different cultures in EFL materials more meaningfully and building schematic frameworks by connecting intercultural learning with learners’ prior knowledge and life experiences (Wu, 2010). As for the shortage of competent teachers, Gonen and Saglam (2012) indicate that most English educators in most EFL contexts avoid or downplay the importance of teaching culture as a part of the language curriculum. This could be attributed to four main causes: first, teachers put more emphasis on the practical aspects of language learning, such as test preparation, grammar, vocabulary, and oral communication (Onalan, 2005); second, institutional demands limit teachers’ capacity and time to effectively address cultural elements in depth in their instructional practices (Cheng, 2012; Liu et al., 2014); third, most teachers in EFL contexts lack proper intercultural experiences and training on how to integrate cultural knowledge into their classroom as well as find an effective way to assess the changes in students’ intercultural competence and attitude after the instruction (Gonen & Saglam, 2012); lastly, intercultural communication occasionally involve controversial topics that learners and teachers from certain cultural backgrounds may find it offensive or inappropriate to address in the educational settings (Sercu, 2005; Young & Sachdev, 2011).

As can be clearly seen in the literature review, while research in English language teaching has stressed the importance of integrating intercultural education as part of language classrooms, factors such as culturally homogeneous classroom, ineffective pedagogies, lack of appropriate materials, and shortage of competent teachers still prevent most EFL students from developing intercultural competence and thereby reducing their overall English learning experiences. Therefore, the aim of this project is twofold: first, it attempts to help address these issues by creating an experiential intercultural English curriculum and investigate how it can affect adolescent EFL student’s intercultural development, motivation to learn English, and proficiency outcome; second, as the literature review shows, it is expected that the results of this research can enrich current studies regarding the effectiveness of ICC in language education that are primarily conducted with adult English learners (e.g., Chao, 2014; Cheng, 2012; Liu, 2017; Tran & Duong, 2018), so that EFL teachers in the middle schools settings can have the pedagogical recourses to draw upon when designing similar curricula.

## Methods

### Research design and procedure

We used a quasi-experimental design to conduct this research to determine whether incorporating ICC into an EFL curriculum affects teenage EFL students’ L2 motivation, intercultural awareness, and English proficiency. To ensure the reliability and validity of the research instruments, in the pilot study, we randomly selected 139 students in the school and used exploratory factor analysis (EFA) to extract the psychometric properties underlying Thai EFL middle school students’ L2 motivations for and attitudes toward learning about foreign cultures and English.

After producing two rating scales, in the formal study, we used the pre-test-post-test control group design by randomly sampling two eighth-grade classes in the participating school, with one class designated as the experimental group (N = 31) and the other as the control group (N = 28). Since this was a quasi-experiment, two classes were randomly selected for this research. For the experimental group, students joined an 8-week cross-cultural curriculum that was instructed in English by a Taiwanese teacher of English (one of the researchers), who met with the class once a week, and a local teacher, who instructed regular English classes in the students’ first language five times a week. As for the control group, they did not receive extra interculturally embedded English courses (instructional treatment); instead, they received regular English courses instructed by another local teacher in their first language. Both groups were asked to fill out two rating scales and take a first Cambridge English examination as their pre-test. After the 8-week period, both groups were asked again to fill out two rating scales and take a second Cambridge English exam as their post-test. Then, the researcher collected the data from both groups through students’ self-assessments of their perceived L2 motivation and intercultural competence, the two test scores of Cambridge English exams, and evaluation questionnaires from the experimental group.

### Research setting

This research project was conducted at a secondary school in Chiang Mai, Thailand. Typically, Thai secondary school has five English classes every week, with each class lasting for 45–50 min, and most English classes are conducted by certified Thai teachers in the Thai language. When this project was conducted, schools were shut down due to the surge in confirmed cases of COVID-19, and all students and teachers were forced to adapt to virtual classrooms and maintain their daily class schedule through Google Meet. As a result of this transition, all the instructions and assessments were implemented in online settings. In addition to the sudden changes in the instructional modes, language barrier between the Taiwanese instructor (who is also one of the researchers) and the Thai students was also a concern. Since the main instructor of this curriculum did not share a common first language with the students, two senior Thai English teachers in the school were present in every class meeting to facilitate the cross-cultural communication and interaction between the instructor and the students.

### Participants

The total participants in this study included 198 adolescent EFL students, with 139 selected for the pilot study and 59 for the formal study. All the students were in the same school. Most of the students in the formal study were at a basic level of proficiency (A1), which was determined on the basis of the Cambridge English exam they took in the pre-test and the teachers’ report. Detailed information regarding the students’ experiences with the use and learning of English is displayed in Table 1. As for the two Thai English teachers involved in this study, both of them were native speaker of Thai and were highly proficient in English. They held university degrees in English and receive teaching certifications awarded by the Ministry of Education of Thailand to be qualified for the teaching positions in the school.

**Table 1:** Students’ general information

	Experimental group (N = 31)	Control group (N = 28)
*Gender*		
Female	20 (64.5%)	15 (53.6%)
Male	11 (35.5%)	13 (46.4%)
*Afterschool English Training*		
No	11 (35.5%)	10 (35.7%)
Yes	20 (64.5%)	18 (64.3%)
*Travel abroad experience*		
Never	21 (67.7%)	23 (82.1%)
1–3 times	7 (22.6%)	5 (17.9%)
4 times or more	3 (9.7%)	0 (0%)
*Exchange experience*		
Never	28 (90.3%)	26 (92.9%)
1–3 times	2 (6.5%)	2 (7.1%)
4 times or more	1 (3.2%)	0 (0%)
*Study abroad experience*		
Never	29 (93.5%)	28 (100%)
Less than half year	0 (0%)	0 (0%)
Half to 1 year	0 (0%)	0 (0%)
Over 1 year	2 (6.5%)	0 (0%)
*Experience of interacting with foreign students or teachers*		
No	24 (77.4%)	21 (75%)
Yes	7 (22.6%)	7 (25%)

### ICC course design using the principles of the backward design model

The backward design model is an instructional framework for designing courses and curricula proposed by Wiggins and McTighe (2005). The model has received growing attention in the field of foreign language education as guidance for language educators to better align the assessment with their instructional practices in the classroom. It is particularly suitable for output- and proficiency-based language teaching as it requires the teacher to focus on what students will ultimately be able to do with the target language rather than simply knowing about its structures (Adair-Hauck et al., 2013).

*Step 1 Identify desired results* Based on the literature review, we identified four major learning goals that are suitable for teenage EFL learners before proceeding with the assessment and course planning:

1. Students will be more willing to respect and appreciate the cultures and traditions of others in intercultural communication settings.
2. Students can use beginner English vocabulary and sentences to interpret and explain a reading texts of an event from another culture and to compare it to their own culture.
3. Students’ motivation to learn English as a communication tool in acquiring new knowledge of cultural practices will be enhanced.
4. Students’ English language competence will improve as a result of intercultural language learning.

*Step 2 Determine acceptable evidence* After identifying the learning goals, the researchers proceeded to design the assessment plans. Since successful intercultural communication requires more output-based language ability, in addition to using the Cambridge English exam to assess whether students’ English ability improves before and after the instruction as a proof of summative assessment, a formative assessment of students’ final oral presentation task was included to examine learners’ learning outcomes. Also, the use of the two rating scales allows researchers to gain more comprehensive insight into the effects of this curriculum design on adolescent students’ progress and attainment.

*Plan learning experiences and instruction.* This experiential intercultural language curriculum was integrated into the regular English classes that the school administered, meaning that the class selected for this teaching experiment would use one extra hour to participate in the curriculum designed by the researchers. The syllabus for each lesson’s topic is provided in Table 2. The name of this 8-week intercultural English curriculum was “A Rice and Culture Tour in Taiwan and Thailand.” The contents of the curriculum were taken from diverse real-world settings and covered a range of discussions about different foods made of rice and their relationship to the cultures and traditions of both countries. To make it a comprehensible input and elicit students’ awareness in the language learning process (Krashen, 1985; Schmidt, 1995, 2001), the researchers adapted the language used in those materials to the students’ English proficiency and designed appropriate comprehension check and interactive activities throughout the course to scaffold and engage the learners.

**Table 2:** Syllabus of “A Rice and Culture Tour in Taiwan and Thailand”

Course outline and topics
1. Introduction—who we are & lecture theme and purpose; introduction to Taiwan
2. Topic 1: Main types of rice in Thailand and Taiwan
3. Topic 2: Rice festivals in Taiwan 1: Taiwanese food made up of sticky rice/compare and contrast rice-related traditions in Taiwan and Thailand
4. Topic 3: Rice festivals in Taiwan 2: Dragon Boat Festivals
5. Topic 4: Rice festivals in Thailand: Royal Plowing Ceremony
6. Topic 5: Food Gallery Project preparation; brainstorming topics (connecting everyday practices related to the rice culture)
7. Mock presentation and presentation skills dissection (template and key vocabulary)
8. Final Food Gallery Project presentation (summative assessment, and each group receives a holistic score with only one chance)

In the first five class sessions, each class consisted of topic-based discussions and a language focus. Students were exposed to a variety of real-life English materials from YouTube, magazines, and traveling brochures on the foods and culture of Taiwan and Thailand, with a special focus on the similarities and differences of rice culture in these countries. During the class, students were encouraged to use each lesson’s key vocabulary and sentence structures to write and share a mini virtual poster sharing their thoughts about the cultural topics addressed in that class session, and they were asked to conduct a group presentation called the “Food Gallery Project” in lesson 8. In this project, conducted through Google Meet, students were asked to work as a group (5–6 persons per group), and each group had to explain how to make the food and what its cultural meaning was in a mini-presentation. The students were explicitly asked to address when and why Thai people ate that food; they were advised to reference the story, customs, festivals, religions, or personal experiences with the food that they wished to introduce. This project allowed students to demonstrate their oral expression and intercultural competence in English by using their life experiences and cultural heritage. To better prepare the students for this assignment, in lessons 6 and 7, the researchers provided guided instruction to equip them with strategies on how to conduct a successful oral presentation, namely a clear visual aid and the use of proper linguistic and non-linguistic communication strategies. It was expected that through a series of highly interactive activities and diverse forms of assessment, students would have the opportunity to improve the listening, speaking, and writing skills required to develop intercultural competence through interaction with researchers, gain knowledge of foreign cultures and be guided to reflect on their own cultures.

### Generating and validating research instruments

This project utilized four instruments to collect data: teenage EFL learners’ L2 motivation rating scale, teenage EFL learners’ intercultural competence rating scale, two Cambridge English exams, and two course evaluation surveys. As the questions on the two rating scales and course evaluation surveys were originally written in the researchers’ L1 (Chinese), three senior English teachers in Thailand were asked to translate into English and Thai and double-check the accuracy of the translated version.

Based on measurement theory, to ensure the reliability and validity of the two rating scales employed in the current study, the researchers conducted a psychometric testing with EFA as a pilot before the formal study so as to assess whether the constructs measured in the scales were suitable in this research context (Pallant, 2007; Slavec & Drnovšek, 2012). For the teenage EFL learners’ L2 motivation rating scale, the researchers first prepared 24 items by adapting Wu’s (2012) EFL high school students’ motivation inventory. As for the teenage EFL learners’ intercultural competence rating scale, 20 items were prepared by adapting Peng et al.’s (2009) intercultural competence rating scale designed specifically for EFL contexts. In the next stage, 139 students in the participating school, none of whom were involved in the formal study, were invited to fill out the questionnaires. Next, EFA was conducted to investigate the reliability and validity of the scales. Based on the results, in the L2 motivation rating scale, six items were deleted. The final draft of the scale included the following four factors: integrated motivation, self-efficacy toward English, external motivation, and English learning for entertainment, with the Cronbach’s alpha coefficient 0.916 and total variance explained 67.09% (Table 3). For the intercultural competence rating scale, four items were deleted. Based on the literature review and the result of factor analysis, the final draft of the scale contained the following three factors: self-efficacy in intercultural situations (which means one’s self-perceived ability to enact communicative skills in ICC settings), display of intercultural awareness (which entails the construct of awareness), and interest in intercultural knowledge (which includes the constructs of attitude and knowledge), with the Cronbach’s alpha coefficient 0.930 and total variance explained 64.49% (Table 4). These indicate a satisfactory level of reliability and internal consistency of the two rating scales used for the present study (Cronbach, 1951; Nunnally, 1978).

**Table 3:** Factor loading for teenage EFL learners’ L2 motivation (N = 139)

Questions	Factor 1	Factor 2	Factor 3	Factor 4
18. I learn English because I want myself to be fluent in it	.718			
20. I learn English to communicate with foreigners	.824			
21. I learn English because it will help me when I travel abroad	.814			
22. I learn English because I want to make friends with foreigners	.804			
23. I learn English because I want to know more about foreign cultures and traditions	.619			
24. I learn English because I might study abroad in the future	.699			
01. I learn English to get good grades		.703		
09. I learn because I am interested in the language		.659		
10. I am very confident in learning of English		.732		
11. I enjoy the process of learning English a lot		.810		
12. Learning English brings me a sense of achievement		.664		
02. I learn English to pass various English exams			.646	
05. I learn English to get into a good school			.635	
06. I learn English because I don’t want to lag behind my classmates			.669	
07. I learn English to meet teachers’ or parents’ expectations			.771	
17. I learn English to increase my competitiveness			.550	
03. I learn English to help me better comprehend English-related materials (e.g., novels, films, comic books, online games, etc.)				.720
04. I learn English so it will be easier for me to surf the internet (e.g., browsing websites, playing video games, etc.)				.803
Total variance explained: 67.09%; Cronbach’s Alpha: .916

**Table 4:** Factor loading for teenage EFL learners’ intercultural competence (N = 139)

Questions	Factor 1	Factor 2	Factor 3
12. I am confident when communicating with people from different cultures	.638		
14. I believe I will be able to deal with negative feelings in a cross-cultural situation (e.g., being stereotyped or misunderstood)	.767		
15. I believe I can develop my own way of learning a foreign language and its culture	.793		
17. I think I can interact with people from different cultures without feeling anxious	.723		
19. I am aware that my cultural background can affect the way I communicate with others (e.g., Asians tend to be more reserved when expressing opinions, whereas Westerners are generally more straightforward)	.573		
20. I am aware that when I get a response from someone from a different cultural background, their ways of responding and communicating reflect the value system of their culture	.615		
08. I like to learn about the differences between my language and culture and those of others		.570	
10. I will properly adjust my attitudes and behaviors when interacting with someone from different cultural backgrounds		.845	
11. I will properly interact with people from different cultural backgrounds to become a global citizen		.723	
16. I will properly prepare before interacting with people from different cultural backgrounds (e.g., familiarizing myself with the basic geography and location of their country)		.643	
18. I don’t see an individual’s behavior as representative of the culture in which he or she is situated (e.g., all Westerners are independent; all Asians are good at math and science)		.695	
01. I like to learn about different cultures around the world			.636
02. I like to interact with people from different cultural and linguistic backgrounds			.577
03. I like to collect artifacts from other cultures (e.g., postcards, paintings, decoration, etc.)			.657
04. I like to learn about other countries’ histories			.827
05. I like to watch shows or programs that introduce foreign cultures or traditions			.867
Total variance explained: 64.49%; Cronbach’s Alpha: .930			

### Data collection and analysis

The researcher collected the data from both groups through students’ self-assessments of their perceived L2 motivation and intercultural competence, the two test scores of the Cambridge English exams, and evaluation questionnaires from the experimental group. A combination of quantitative and qualitative methods was used to obtain evidence of the development of ICC, L2 motivation, and English competence.

For quantitative analysis, IBM SPSS statistical software was used to conduct statistical analysis (means, standard deviation, EFA, and one-way repeated measures analysis of variance [ANOVA]) to identify any statistical differences between the pre-test and post-test of the two affective attributes reflected in the rating scales and students’ test scores on the Cambridge English exam. One-way repeated measures ANOVA also provides researchers with empirical insight into different developmental trajectories and learning outcomes in the two groups by repeatedly measuring and comparing the students who attended the ICC-based English class with those who did not. As for the evaluation survey questionnaires, which are designed to assess the experimental group students’ and teachers’ degree of satisfaction regarding overall course contents, instructional techniques, and interactional experiences; for the assessment of students’ perception (Table 5), items 1 to 11 consist of 5-point Likert-scale questions and analyzed through mean scores, with 5 means strongly agree, 4 means agree, 3 means OK, 2 means disagree, and 1 means strongly disagree. Regarding the assessment of teachers’ perception (Table 6), items 1 to 10 are composed of the same analysis with 5-point Likert-scale questions.

**Table 5:** Survey of the course for students in the experimental group

Questions	5	4	3	2	1
01. The content of the course is not too difficult or too easy for me					
02. The teacher’s instruction is easy to understand throughout the curriculum					
03. The teacher can use a variety of methods to help me understand the contents of the course					
04. I really like this intercultural English curriculum					
05. After this intercultural English curriculum, I learned more about Thailand’s culture					
06. After this intercultural English curriculum, I learned more about Taiwanese culture					
07. After this intercultural English curriculum, I understood the importance of learning English and using it to communicate with others					
08. After this intercultural English curriculum, I became more motivated to learn English					
09. After this intercultural English curriculum, I am less afraid of speaking English					
10. After this intercultural English curriculum, I am more confident in communicating and interacting with foreigners					
11. I hope to take a similar intercultural English curriculum in the future					
12. Please describe what you have learnt the most in this intercultural English curriculum?					
13. Are there any suggestions for this intercultural English curriculum?					

Strongly agree (5); Agree (4); OK (3); Disagree (2); Strongly disagree (1)

**Table 6:** Survey of the course for teachers in the experimental group

Questions	5	4	3	2	1
01.The course contents of this English curriculum are suitable based on Thai students’ English proficiency					
02. The teacher’s instructions are clear and easy to understand for the students					
03. The teacher could use diverse teaching techniques and modes of instruction to let students fully understand the course contents					
04. This web-based English curriculum can help Thai students better understand their own culture					
05. This English curriculum can help Thai students learn Taiwanese culture better					
06. This English curriculum can let students know the importance of learning English and using English to communicate with foreigners					
07. This English curriculum can increase students’ motivation to learn English					
08. Students are more willing to speak English after this online English curriculum					
09. The content, materials, activities, and assessment design of this virtual English curriculum match the objectives of the course design					
10. Thai teachers can apply the topics and content of this English curriculum to their English teaching in the future					
11. What are the most significant differences between this English curriculum and my past teaching experiences?					
12. What kind of suggestions would you like to offer to the teachers and the implementation of this English curriculum?					

Strongly Agree (5); Agree (4); OK (3); Disagree (2); Strongly Disagree (1)

In terms of qualitative analysis, for students’ evaluation survey questionnaire, question 12 and 13 ask the leaners’ responses with open questions regarding what they have learned the most and what can be improved in the instruction; for the teachers’ survey, questions 11 and 12 require two teachers to reflect the most significant differences between the implementation of this curriculum and their past teachings and what can be improved. All results were analyzed through thematic analysis, and the responses or comments were read thoroughly by the researchers, who continually compared respective coded texts and revised them several times before the themes emerged. The response was coded with a cardinal number, such as S1, T1, and so on, for confidentiality and ethical purposes.

## Results

### Teenage EFL students’ L2 motivation

**Fig. 1:**
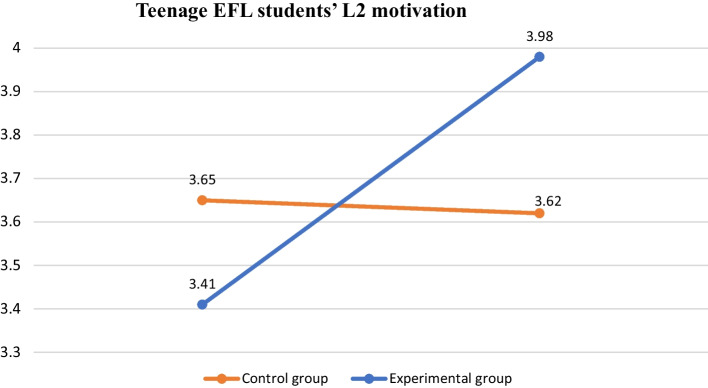
Teenage EFL students' L2 motivation

As can be seen from Tables 7 and 8, the results gathered from the one-way repeated measures ANOVA of students’ self-assessment of their perceived motivation for learning English show a difference between the two groups of students. To be specific, students’ average mean scores of their perceived overall motivation increased from 3.41 to 3.98 in the experimental group, while control group students’ average mean score showed a decreased pattern from 3.65 in their pre-test to 3.62 after the 8-week period. In terms of the factor of integrated motivation, students’ mean scores in the experimental group increased from 3.43 to 4.00, while the mean scores of students in the control group decreased from 3.75 to 3.72. With regard to self-efficacy toward English learning, for students in the experimental group, it grew from 3.29 to 3.80, while it decreased from 3.60 to 3.52 for students in the control group. With regard to external motivation, the mean scores of the students in the experimental group increased from 3.29 to 3.95, and the mean scores of those in the control group slightly decreased from 3.49 to 3.45. Finally, with regard to learning English for entertainment, the scores of students in the experimental group increased from 3.78 to 4.06, and those of students in the control group also slightly increased from 3.91 to 3.95. Overall, through the comparison chart in Fig. 1, it is evident that adolescent EFL students who followed the interculturally embedded English curriculum tended to improve their overall motivation toward English learning compared with those who did not receive cultural-based language instruction after the 8-week data collection period.

**Table 7:** L2 motivation rating scale (experimental group)

Item (N = 31)	Pre-test M (SD)	Post-test M (SD)
Integrated motivation	3.43 (.33)	4.00 (.27)
Self-efficacy toward English	3.29 (.23)	3.80 (.32)
External motivation	3.29 (.39)	3.95 (.35)
Learning English for entertainment	3.78 (.24)	4.06 (.20)
Overall motivation means	3.41 (.24)	3.98 (.29)

**Table 8:** L2 motivation rating scale (control group)

Item (N = 28)	Pre-test M (SD)	Post-test M (SD)
Integrated motivation	3.75 (.55)	3.72 (.50)
Self-efficacy toward English	3.60 (.45)	3.52 (.56)
External motivation	3.49 (.32)	3.45 (.36)
Learning English for entertainment	3.91 (.51)	3.95 (.45)
Overall motivation means	3.65 (.37)	3.62 (.44)

### Teenage EFL students’ intercultural competence

**Fig. 2:**
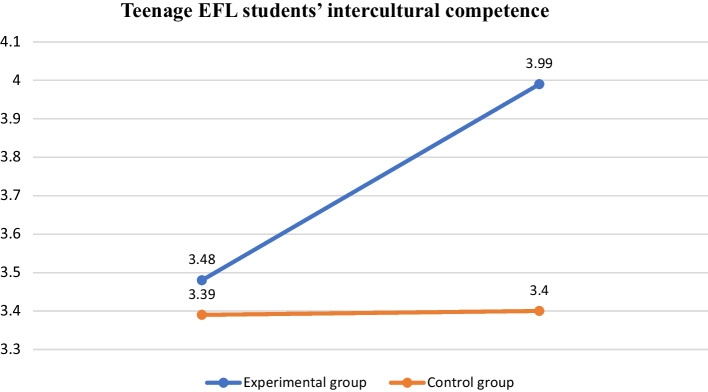
Teenage EFL students’ intercultural competence

As for the students’ self-perceived development of ICC, Tables 9 and 10 show that in the pre-test, students’ overall ICC in the experimental group had average mean scores of 3.48, and these increased to 3.99 in the post-test, while the mean scores of the students in the control group slightly increased from 3.39 in the pre-test to 3.40 after the 8-week instruction. In terms of the factor of self-efficacy in intercultural situations, the students’ mean scores in the experimental group increased from 3.35 to 3.85, while the mean scores of the students in the control group increased from 3.34 to 3.35. With regard to the display of intercultural awareness, the scores of students in the experimental group grew from 3.59 to 4.20, while those of students in the control group decreased from 3.58 to 3.56. With regard to the interest in intercultural knowledge, the mean scores of the experimental group students increased from 3.45 to 4.09, and the mean scores of the students in the control group rose slightly from 3.25 to 3.29. In sum, based on the comparison chart in Fig. 2, it is also evident that students who followed the English curriculum with cross-cultural input tended to improve their intercultural competence compared to those who did not receive any culturally based language instruction after the 8-week data collection period.

**Table 9:** Intercultural competence rating scale (experimental group)

Item (N = 31)	Pre-test M (SD)	Post-test M (SD)
Self-efficacy in intercultural situations	3.35 (.45)	3.85 (.37)
Display of intercultural awareness	3.59 (.52)	4.20 (.40)
Interest in intercultural knowledge	3.45 (.57)	4.09 (.29)
Overall intercultural competence means	3.48 (.30)	3.99 (.41)

**Table 10:** Intercultural competence rating scale (control group)

Item (N = 28)	Pre-test M (SD)	Post-test M (SD)
Self-efficacy in intercultural situations	3.34 (.58)	3.35 (.35)
Display of intercultural awareness	3.58 (.47)	3.56 (.65)
Interest in intercultural knowledge	3.25 (.53)	3.29 (.49)
Overall intercultural competence means	3.39 (.53)	3.40 (.31)

### Teenage EFL students’ English language competence

**Fig. 3:**
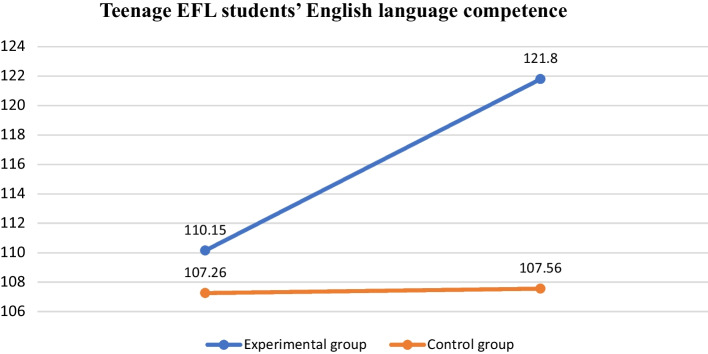
Teenage EFL students’ English language competence

According to Table 11, the results of the Cambridge English exam demonstrate the effectiveness of teenage EFL students’ progress in English language competence, which means that incorporating ICC into English instruction is conducive to EFL learners’ acquisition of English ability. The scores reported in the study conform to the Cambridge English Scale. With respect to the pre-test, the average mean score of students in the experimental group was 110.15, while that of students in the control group was 107.26. Concerning the post-test, the average mean score of students in the experimental group was 121.80, while students from the control group obtained a mean score of 109.56. Together with the comparison chart in Fig. 3, the results suggest that after the 8-week period, students who followed the interculturally based English course showed greater degree of improvement in their English language ability compared with those who did not immerse in an all-English and intercultural-related language classroom.

**Table 11:** Test scores using the Cambridge English Scale

Pre-test		Post-test	
M	SD	M	SD
*Experimental group*			
110.15	9.20	121.80	10.10
*Control group*			
107.26	13.15	109.56	10.11

### Students’ perceptions of the course

To triangulate the data and obtain a more in-depth and nuanced understanding of the effectiveness of this course design and the kind of trajectories that learners in the experimental group followed, both the quantitative and qualitative data from the two post-curriculum questionnaires used to collect students’ and teachers’ responses were analyzed.

For the survey with students in the experimental group (Table 12), based on the quantitative finding, the average mean scores of the first 11 questions were close to 4, especially for Questions 6 and 7, indicating a rather strong agreement with English learning experiences and progress in the development of intercultural knowledge and competence. Questions 1, 4, and 5, which received mean scores of 3.5 and 3.7, respectively, show moderate agreement among most students because they demonstrated perceived difficulty toward certain parts of the content and language that the instructor used in class.

**Table 12:** Survey of the course for students in the experimental group (N = 31)

Questions	M	SD
01. The content of the course is not too difficult or too easy for me	3.5	0.7
02. The teacher’s instruction is easy to understand throughout the curriculum	3.8	0.6
03. The teacher can use a variety of methods to help me understand the contents of the course	3.8	0.6
04. I really like this intercultural English curriculum	3.7	0.6
05. After this intercultural English curriculum, I learned more about Thailand’s culture	3.7	0.6
06. After this intercultural English curriculum, I learned more about Taiwanese culture	4.0	0.8
07. After this intercultural English curriculum, I understood the importance of learning English and using it to communicate with others	4.0	0.7
08. After this intercultural English curriculum, I became more motivated to learn English	3.8	0.6
09. After this intercultural English curriculum, I am less afraid of speaking English	3.8	0.7
10. After this intercultural English curriculum, I am more confident in communicating and interacting with foreigners	3.9	0.7
11. I hope to take a similar intercultural English curriculum in the future	3.8	0.8
12. Please describe what you have learnt the most in this intercultural English curriculum?		
13. Are there any suggestions for this intercultural English curriculum?		

Strongly agree (5); Agree (4); OK (3); Disagree (2); Strongly disagree (1)

The qualitative data analysis of Question 12 shows that 80% of the participants thought the course would help them increase cross-cultural awareness and obtain a deeper understanding of the differences between their own cultures and foreign cultures. Sample responses are as follows:

I have learned a lot about the differences between Thailand and Taiwan. I liked how teacher Leo wrote several traditions in Taiwan on Padlet and asked us to think whether there are similar ones in our country’s culture or not. (S3)

In addition, 70% mentioned that they benefited greatly from listening and speaking practice:

Because teacher Leo did not speak Thai, the only way we could talk to him was in English. He taught us a lot of new vocabulary and phrases that I did not know before, and he taught us how to use those words to express ideas related to culture. (S16)

Furthermore, 81% thought the final Food Gallery Project provided them with an intense but valuable opportunity to collaborate with other peers and produce their group presentation—a previous experience that many of them did not have:

I had not had many chances to speak English in the past, but teacher Leo asked us to prepare a food gallery project. Even though I am still afraid of speaking English in front of so many people, I like to work with classmates together and finally be able to present my parts. (S17)

As for Question 13, which deals with how this course can be improved, 40% of students mentioned that it could be better if the teacher spoke more slowly so that the students can comprehend the materials or instructions and perhaps engage more in the instruction:

At the beginning, teacher Leo spoke very fast. Because I did not know every word on the PPT and the YouTube video about Taiwan, I had to think about it and check the words. Also, it is an online class, and I don’t really like to ask questions when teachers are speaking. (S19)

### Teachers’ perceptions of the course

In terms of the survey with two senior English teachers participating in the experimental group (Table 13), Questions 1–10 were analyzed through quantitative methods, and Questions 11 and 12 through qualitative methods. As the table demonstrates, teachers were generally satisfied with the overall design of the course in terms of the instructional modes, content selections, and the applicability to their future English teaching agenda.

**Table 13:** Survey of the course for teachers (N = 2)

Questions	M	SD
01.The course contents of this English curriculum are suitable based on Thai students’ English proficiency	3.5	0.7
02. The teacher’s instructions are clear and easy to understand for the students	4.5	0.7
03. The teacher could use diverse teaching techniques and modes of instruction to let students fully understand the course contents	3.5	0.7
04. The web-based English curriculum can help Thai students better understand their own culture	4.5	0.7
05. The English curriculum can help Thai students learn Taiwanese culture better	5	0
06. The English curriculum can let students know the importance of learning English and using English to communicate with foreigners	4	0
07. The English curriculum can increase students’ motivation to learn English	4.5	0.7
08. Students are more willing to speak English after this online English curriculum	3.5	0.7
09. The content, materials, activities, and assessment design of this virtual English curriculum match the objectives of the course design	5	0.0
10. Thai teachers can apply the topics and content of this online English curriculum to their English teaching in the future	4.5	0.7
11. What are the most significant differences between this English curriculum and my past teaching experiences?		
12. What kind of suggestions would you like to offer to the teachers and the implementation of this English curriculum?		

Strongly agree (5); Agree (4); OK (3); Disagree (2); Strongly disagree (1)

Some qualitative evidence of teachers’ opinions about this course is as follows:

This is an all-English curriculum that focuses mostly on the topic of cross-cultural learning and communication strategies, which is helpful in helping students understand and develop cultural sensitivity and communicative competence. The idea of using a backward design is interesting because teachers can have a clear sense of what kind of competencies to teach before making a classroom plan. (T1)

This English curriculum also provides experiential cross-cultural opportunities for students to use English to talk with teachers from Taiwan and taste Taiwanese rice, so that the students have a deep impression of the lesson. In addition, one class session focuses on oral presentation skills, and the teacher provides a template that is so operationalizable for lower-intermediate students like my class. (T2)

However, like some students’ responses regarding English-medium instruction, Questions 1, 3, and 8, which received a slightly lower mean score of 3.5, reflected teachers’ opinion that some contents and language used were indeed too difficult for the participants’ English level; further, for students whose English was already poor, such English immersion-based instruction could not elevate their motivation and engagement in the classroom.

Some content is rather difficult. I hope teachers can provide more warm-up activities before teaching each lesson to prepare students in our contexts. (T1)

To improve the listening and oral expression skills of the students, repetition techniques and feedback can be used more for dealing with situations where lower-level students do not understand the target language. (T2)

Despite some drawbacks of this all-English course design, generally speaking, with Thai teachers’ understanding of their students’ classroom cultures, learning styles, and proficiency level, the concepts and materials involved in this cross-cultural learning are still of immense usefulness for teachers of English in Thailand to make adjustments and apply them to other teaching contexts in the future.

## Discussion

### Research question 1: Does the ICC-based English curriculum affect adolescent EFL learners’ L2 motivation?

The results obtained from the rating scale of EFL learners’ perceived motivation for learning English and post-curriculum questionnaires show a different pattern of motivation change between two groups of students after the 8-week, ICC-based English instruction. These findings indicate that the inclusion of cultural input in foreign language classrooms can not only improve adolescent EFL learners’ motivation for English learning in general, but it can also engage middle school students with the learning process in the public-school setting in an EFL context. The possible explanation of such positive effects on students’ L2 motivation is that through experiential cultural learning, students are provided with opportunities not only to experience how linguistic knowledge (e.g., grammar, vocabulary, and syntax) works in real-world communication settings but also to use English as a means of communication to interpret and understand new knowledge that is represented in their target language. This is evident in the similar pattern of increase in the four components of adolescent EFL learners’ motivation discovered in our quantitative analysis. In other words, students in the experimental group realize that the learning of a new language is no longer about mindlessly receiving the linguistic knowledge in a decontextualized manner, as seen in most EFL classrooms, but through cross-cultural interaction with foreign teachers and other tangible materials in which the target language features, and cultural knowledge is meaningfully presented in a connected discourse. As a result, once the learners can sense the meaning of how the target language works in real-life settings such as intercultural communication situations, their motivation for learning English is enhanced because they are aware that English would allow them to further explore how the target language operates in the context of intercultural communication (Brown, 2012; Trappes-Lomax, 2004).

However, in terms of the drawback of this curriculum design, questions arise as to whether immersing teenage EFL students in an all-English environment is conducive to all students’ English learning experiences or outcomes. Even though the data from the quantitative analysis indicate that students exposed to culturally related language classrooms tend to be more motivated to engage in English learning compared with those without, the results from the qualitative analysis show that students with a lower level of proficiency may not benefit significantly from this kind of instruction. From the perspective of second language acquisition, if the difficulty of course contents is far beyond the learners’ proficiency level, the notice and meaningful processing mechanism is unlikely to be stimulated (Krashen, 1985; Schmidt, 1995, 2001); under such circumstances, for students who did not have many English and intercultural experiences, such learners may gain a sense of detachment from classroom activities because the language used throughout the course prevents them from delving into intercultural discourse and the acquisition of intercultural knowledge. They may even feel anxious when teachers try to initiate intercultural-related questions in English due to a lack of proficiency to comprehend such information. Furthermore, the online nature of this course, to some extent, could have impacted the success of this project because teachers do not have face-to-face interactions with the students. The online delivery can also present some difficulties as teachers cannot easily monitor students’ real-time behaviours or comprehension. As the sense of detachment and anxiety accumulates, learners with low English proficiency would become less engaged in the learning process as the overall contents are too opaque for them, and a sense of frustration in this learning process results in less effective learning.

### Research question 2: Does the ICC-based English curriculum help adolescent EFL learners develop intercultural competence?

The quantitative findings from the rating scale of adolescent EFL students’ intercultural competence and two post-curriculum surveys indicate that the incorporation of cultural knowledge into English learning is effective in elevating EFL learners’ intercultural knowledge and is feasible for middle school English classroom settings. For students in the experimental group, all three components underlying adolescent EFL student’s intercultural competence (self-efficacy in intercultural situations, display of intercultural awareness, and interest in intercultural knowledge) demonstrate a similar pattern of increase compared with students in the control group, who show a decrease of their display of intercultural awareness and of affection toward intercultural knowledge and only a slight increase of self-efficacy in intercultural situations. One of the possible explanations is that most students in the experimental group agreed that they found the contents of this course informative and provided them with a different learning channel to learn about new cultures by making use of the linguistic knowledge in intercultural communication settings and engaging in real cross-cultural interaction, and this can be seen as an improvement of the skills aspects of ICC (Liu, 2017; Peng et al., 2009). Specifically, the presence of the researchers as the main instructors of English in this context provides learners with an invaluable opportunity to discuss the cultural differences between the two countries through the use of real-world materials that enrich the learning process and foster the outcome of students’ learning—especially for most EFL middle school students who have limited experience in using a foreign language in an intercultural setting as the background survey demonstrates.

Another significant finding of this study is that students’ curiosity and willingness to learn cross-cultural knowledge was elicited as a result of this course design. Based on the data obtained from the questionnaires from both the students and teachers involved in the experimental group, the design of the culturally related English curriculum in this study is capable of overcoming the challenges of including intercultural language teaching in EFL classrooms; in other words, as the lack of diverse cultural materials in a culturally and linguistically homogeneous classroom can make it difficult for learners to acquire and make use of ICC strategies in EFL contexts (Cheng, 2012; Tran & Duong, 2018), this course design provides an experiential learning environment for teenage EFL students to develop reflective awareness of cultural elements through the interaction with the instructors and diverse culturally related materials in classroom activities, particularly for those who had limited experiences interacting with foreigners. In addition, most students displayed an interest in knowing more about the Taiwanese people’s ways of life and what rice means to them in their daily lives. Some students who were more outgoing, proficient in English, or had more experiences in interacting with people from different cultures even demonstrated better ability to compare and contrast their own cultures with the foreign culture and apply this knowledge in the communicative tasks assigned by the instructors in diverse ways of written and spoken productions in English—in this case, the poster writings on the Padlet and the final food gallery presentation project. Through this experiential and communicative course design, adolescent EFL students are endowed with an environment that promotes comparisons between different cultures by connecting intercultural learning with learners’ prior knowledge and life experiences with the learning of culture in English classrooms, regardless of what degree of intercultural experiences they have had before joining this project (Diaz-Rico, 2013; Quiocho & Ulanoff, 2009; Wu, 2010).

### Research question 3: Does the ICC-based English curriculum increase adolescent EFL learners’ language competence?

The results from the pre-test and post-test of the Cambridge English exam show that students in the experimental group have more improvement in English competence than those in the control group, which suggests that the inclusion of intercultural communication-related content with English language learning can facilitate young EFL students’ acquisition of English. This can be seen in this course design; throughout the course of the instruction, a variety of language features such as vocabulary, sentence structures, and formulaic sequences that are typical of expressing cultural information were instructed along with communicative-based tasks for learners’ to immediately use those features in meaningful contexts. In addition, since the main instructors of this course design had different linguistic and cultural backgrounds, the students were expected to use the target language to participate in class activities, such as seeking clarification from the teachers, understanding the instructions from the instructors, and completing assignments. The researchers also assigned students into groups, where more proficient learners serve as the leaders or peer mentors to help learners with low proficiency be more emotionally and linguistically prepared for classroom discussions, group work, and comprehension-based activities. Such collaborative pedagogy echoes earlier studies concerning the sociocultural view of second language acquisition, in which acquisition best occurs when students are engaged in collaboration with either their peers or instructors (Firth & Wagner, 2007; Lantolf, 2011; Vygotsky, 1986). From the cognitive aspects of second language acquisition, the possible explanation of such positive effects on students’ L2 proficiency is that in this experiential cultural learning, the researchers constantly direct students’ attention to how linguistic knowledge (i.e., grammar, vocabulary, and syntax) operationalizes in real-world communication settings (real-world intercultural materials) but to using English as a means of communication to process new knowledge that is represented in their target language. In this way, teachers can ensure that while engaging in intercultural communicative tasks and materials, students are still provided with “the type of negotiated interaction and meaningful input and to produce the target language in response to the input, and to receive feedback on learner production” (Nassaji & Fotos, 2011, p. 89).

## Conclusion

The qualitative and quantitative data in this study demonstrated that utilizing an ICC-based English curriculum would stimulate EFL secondary school students’ L2 motivations for and attitudes toward learning about foreign cultures as well as increase students’ English competence. Specifically, using a backward design model to construct classroom-based experiential learning may be helpful to equip teenage EFL learners with both ICC and English ability to become not only fluent in a foreign language but also to function appropriately and effectively in a globalized context. Furthermore, this teaching model also demonstrates its potential to help address the critical issues that hinder adolescent EFL students’ learning of intercultural knowledge. In other words, secondary school EFL teachers can adapt this model to the teaching of different intercultural content or different foreign languages to foster students’ learning. This kind of curriculum design can also be supplemental to the cultural contents in textbooks to help overcome the issues of the lack of diverse cultural inputs in most foreign language textbooks and a culturally homogeneous learning environment. Instead, teenage EFL students are provided with a space where comparisons and contrasts between different cultures are more meaningful and built to connect intercultural learning with learners’ prior knowledge and life experiences.

However, since this teaching experiment is conducted in a single EFL classroom in Thailand, to make this teaching model more applicable to similar settings and specifically for adolescent students, the following suggestions are made for future research: (1) this model should be tested at various proficiency levels in different EFL contexts, with a particular focus on adolescent English learners with different first language and cultural backgrounds in secondary school settings; (2) inviting other international students who are both native or non-native speakers of English and of the same age as the participants to enhance more meaningful cross-cultural interaction and foreign language learning experience; and (3) studying periods could be lengthened to compensate for EFL students’ limited exposure to the target language and cultural environment.

## Data Availability

The datasets used and/or analyzed during the current study are not publicly available due to protection of the participants’ confidentiality but are available from the corresponding author upon reasonable request.

## Abbreviations

ANOVA: Analysis of variance
CLT: Communicative language teaching
EFL: English as a foreign language
EFA: Exploratory factor analysis
ICC: Intercultural communicative competence
L1: First language
L2: Second language
